# Ischaemic stroke in women with atrial fibrillation: temporal trends and clinical implications

**DOI:** 10.1093/eurheartj/ehae198

**Published:** 2024-04-12

**Authors:** Konsta Teppo, K E Juhani Airaksinen, Jussi Jaakkola, Olli Halminen, Birgitta Salmela, Elis Kouki, Jari Haukka, Jukka Putaala, Miika Linna, Aapo L Aro, Pirjo Mustonen, Juha Hartikainen, Gregory Y H Lip, Mika Lehto

**Affiliations:** Heart Centre, Turku University Hospital and University of Turku, Turku, Finland; Heart Centre, Turku University Hospital and University of Turku, Turku, Finland; Heart Centre, Turku University Hospital and University of Turku, Turku, Finland; Department of Industrial Engineering and Management, Aalto University, Espoo, Finland; Department of Internal Medicine, Heart Center, Päijät-Häme Central Hospital, Lahti, Finland; Faculty of Medicine, University of Helsinki, Helsinki, Finland; Faculty of Medicine, University of Helsinki, Helsinki, Finland; Neurology, Helsinki University Hospital and University of Helsinki, Helsinki, Finland; Department of Industrial Engineering and Management, Aalto University, Espoo, Finland; Department of Health and Social Management, University of Eastern Finland, Kuopio, Finland; Heart and Lung Center, Helsinki University Hospital and University of Helsinki, Helsinki, Finland; Heart Centre, Turku University Hospital and University of Turku, Turku, Finland; Heart Center, Kuopio University Hospital and University of Eastern Finland, Kuopio, Finland; Liverpool Centre for Cardiovascular Science at University of Liverpool, Liverpool John Moores University and Liverpool Heart & Chest Hospital, Liverpool, UK; Department of Clinical Medicine, Danish Center for Health Services Research, Aalborg University, Aalborg, Denmark; Department of Internal Medicine, Jorvi Hospital, HUS Helsinki University Hospital and University of Helsinki, Helsinki, Finland

**Keywords:** Atrial fibrillation, Female sex, Women, Ischaemic stroke, Temporal trends

## Abstract

**Background and Aims:**

Female sex has been linked with higher risk of ischaemic stroke (IS) in atrial fibrillation (AF), but no prior study has examined temporal trends in the IS risk associated with female sex.

**Methods:**

The registry-linkage Finnish AntiCoagulation in Atrial Fibrillation (FinACAF) study included all patients with AF in Finland from 2007 to 2018. Ischaemic stroke rates and rate ratios were computed.

**Results:**

Overall, 229 565 patients with new-onset AF were identified (50.0% women; mean age 72.7 years). The crude IS incidence was higher in women than in men across the entire study period (21.1 vs. 14.9 events per 1000 patient-years, *P* < .001), and the incidence decreased both in men and women. In 2007–08, female sex was independently associated with a 20%–30% higher IS rate in the adjusted analyses, but this association attenuated and became statistically non-significant by the end of the observation period. Similar trends were observed when time with and without oral anticoagulant (OAC) treatment was analysed, as well as when only time without OAC use was considered. The decrease in IS rate was driven by patients with high IS risk, whereas in patients with low or moderate IS risk, female sex was not associated with a higher IS rate.

**Conclusions:**

The association between female sex and IS rate has decreased and become non-significant over the course of the study period from 2007 to 2018, suggesting that female sex could be omitted as a factor when estimating expected IS rates and the need for OAC therapy in patients with AF.

## Structured Graphical Abstract

**Structured Graphical Abstract:**
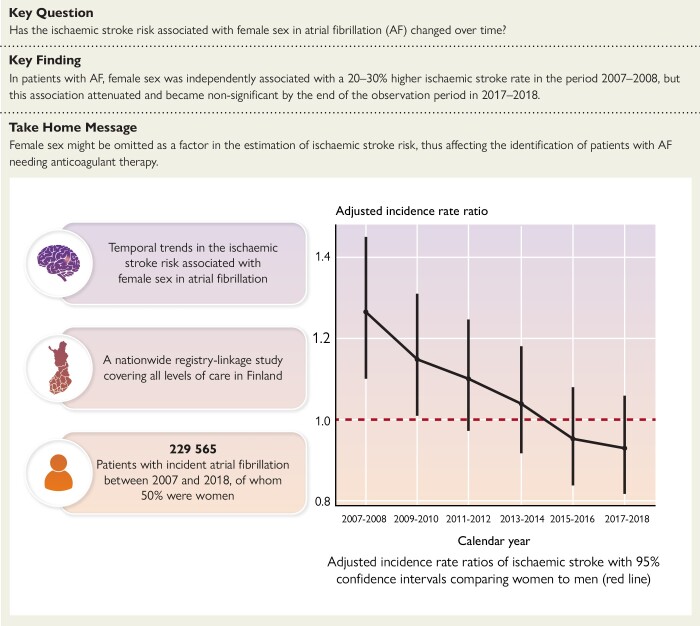
Temporal trends in the association between female sex and ischaemic stroke among patients with atrial fibrillation in Finland from 2007 to 2018.

**See the editorial comment for this article ‘Taking the sex out of atrial fibrillation’, by D. Brieger and B. Freedman, https://doi.org/10.1093/eurheartj/ehae256.**

## Introduction

Atrial fibrillation (AF) is the most common cardiac arrhythmia, affecting up to 5.2% of the adult population.^1^ It is a major cause of ischaemic stroke (IS), with the risk of stroke varying considerably among individuals based on their comorbidities and other characteristics.^2,3^ Accurate stratification of stroke risk and identification of individuals who would benefit from oral anticoagulant (OAC) therapy for stroke prevention is essential in managing patients with AF.

Women with AF are usually older and present with a higher burden of comorbidities than men, reflecting in higher IS rates in women.^1,4,5^ Additionally, female sex has been independently associated with risk of IS already in the early OAC trials and thereafter in observational studies.^6,7^ In 2010, the CHA_2_DS_2_-VASc stroke risk score, which included female sex as a factor, was proposed and subsequently adopted into international guidelines on AF management.^2,8–10^ However, the IS risk linked to female sex has an age dependency, and it is more a risk modifier in the presence of other IS risk factors.^11–13^ The connection between female sex and thromboembolic risk is believed to stem from a variety of factors related to both biologic sex and sociocultural gender, encompassing hormonal, structural, endocrine, lifestyle, and social components, but the precise pathophysiological mechanisms of this phenomenon have remained incompletely understood.^14–16^ Additionally, inequities in cardiovascular care have been shown to partly explain the higher stroke rates in women with AF.^13^

While the interplay between female sex and IS risk has been extensively studied, there is a paucity of information regarding the temporal trends in their association in patients with AF. Considering the intricate pathways underlying the elevated stroke risk in women, the advances in the optimal management of other traditional stroke risk factors, and the historical trends in gender inequalities in both health and broader societal contexts, we hypothesized that the impact of female sex on the risk of IS in patients with AF has also changed over time. Thus, we conducted a nationwide cohort study covering all patients with incident AF in Finland between 2007 and 2018 to explore the temporal trends in IS risk associated with female sex.

## Methods

The Finnish AntiCoagulation in Atrial Fibrillation (FinACAF) study (ClinicalTrials Identifier: NCT04645537; ENCePP Identifier: EUPAS29845) is a nationwide retrospective cohort study that includes patients diagnosed with AF at all levels of care in Finland from 2004 to 2018.^17^ Patients were identified using all available national healthcare registers, including hospitalizations and outpatient specialist visits (HILMO), primary healthcare (AvoHILMO), and the National Reimbursement Register maintained by the Social Insurance Institute (KELA). The cohort inclusion criterion was an International Classification of Diseases, Tenth Revision (ICD-10), diagnosis code of I48, encompassing AF and atrial flutter, collectively referred to as AF, recorded between 2004 and 2018. Exclusion criteria were permanent emigration abroad before 31 December 2018 and age below 20 years at AF diagnosis. The present sub-study was conducted within a cohort of patients with incident AF from 2007 to 2018, established in previous studies of the FinACAF cohort.^18–20^ In this cohort, to include only patients with newly diagnosed AF, a washout period was applied by excluding those with a recorded AF diagnosis during 2004–06, because the medical history of <2 years was considered too short to exclude the presence of a prior AF diagnosis. Additionally, to ensure capturing the true initiation of OAC therapy and exclusion of patients with prior AF, those with a fulfilled OAC prescription during 2004–06 or within a year before the first AF diagnosis were excluded (see Supplementary data online, *Figure S1*).

The follow-up was primarily analysed with two separate approaches. In both methods, follow-up started from the initial AF diagnosis. In the first approach, we analysed the overall cohort with the entire follow-up continuing until the first observed IS event, death, or 31 December 2018, whichever came first. In this approach, analyses were adjusted for OAC use. Additionally, since it is the non-anticoagulated IS rate that drives the clinical decision-making of stroke prevention with OACs, in the second approach, we concentrated solely on the follow-up time without OAC therapy. In these analyses, follow-up continued only until the first OAC purchase, the first IS event, death, or 31 December 2018, whichever came first. The latter approach has been previously suggested for estimating event rates in untreated populations.^21,22^

Patients often develop new comorbidities after the initial diagnosis of AF, impacting the risk of IS and making their IS risk a dynamic process.^23^ To address potential bias associated with changing IS risk due to increasing age and incident comorbidities over a longer follow-up period, we also conducted analyses with a follow-up period restricted to a maximum of 1 year after the initial AF diagnosis, i.e. continuing until the occurrence of the first IS event, death, 31 December 2018, or a maximum of 1 year after AF diagnosis. Moreover, in these analyses of 1-year follow-up, we divided patients into categories based on their baseline stroke risk (low risk, CHA_2_DS_2_-VASc score 0 in men and 1 in women; moderate risk, CHA_2_DS_2_-VASc score 1 in men and 2 in women; high risk, CHA_2_DS_2_-VASc score > 1 in men and >2 in women; very high risk, CHA_2_DS_2_-VASc score > 2 in men and >3 in women). The analyses by risk category utilized the 1-year follow-up. Of note, the quality of the primary healthcare register (AvoHILMO) has been shown to be inferior to that of the well-validated hospital care register (HILMO), and patients with AF have been identified from reimbursement data increasingly after the introduction of direct OACs.^24^ To address potential selection bias arising from these factors, we conducted additional sensitivity analyses focusing exclusively on patients with AF identified from the hospital care register (HILMO), aligning with the approach commonly employed in previous studies on IS rates in patients with AF.^11,21,25^ Data on baseline comorbidities were obtained from the aforementioned healthcare registers from all levels of care. The definitions of baseline comorbidities are presented in Supplementary data online, *Table S1*.

### Outcomes

In patients without prior IS before or at the same date as the first AF diagnosis, IS event was considered to occur on the first date of a recorded I63 or I64 ICD-10 diagnosis code in the hospital care register after the cohort entry. In patients with prior IS before or at AF diagnosis, the event was considered to occur on the date of the first new hospitalization with I63 or I64 ICD-10 code as the main diagnosis with at least a 90-day gap from the prior event, which had occurred before AF diagnosis. Only IS diagnoses from the hospital register were included to ensure that the event of interest was truly major and clinically relevant.

### Study ethics

The study protocol was approved by the Ethics Committee of the Medical Faculty of Helsinki University, Helsinki, Finland (nr. 15/2017), and received research permission from the Helsinki University Hospital (HUS/46/2018). Respective permissions were obtained from the Finnish register holders (KELA 138/522/2018, THL 2101/5.05.00/2018, Population Register Centre VRK/1291/2019-3, Statistics Finland TK-53-1713-18/u1281, and Tax Register VH/874/07.01.03/2019). Patients’ personal identification numbers were pseudonymized, and the research group received individualized but unidentifiable data. Informed consent was waived due to the retrospective registry nature of the study. The study conforms to the Declaration of Helsinki as revised in 2013.

### Statistical analyses

We estimated incidence rates and incidence rate ratios (IRRs) for IS using the Poisson regression model with a Lexis-type data structure based on three time scales: follow-up time from AF diagnosis, calendar year in 2-year intervals, and age.^26^ This statistical method was chosen to compute IS rates for each calendar year interval and to account for patients’ age increasing during the relatively long observation period between 2007 and 2018. With this method, patients’ age at each point of follow-up was calculated. Subsequently, age was split into 5-year intervals and considered as a categorical variable in the analyses. Correspondingly, calendar years were divided into 2-year intervals. The adjusted analyses included the following variables: age, calendar year, heart failure, hypertension, diabetes, prior IS or transient ischaemic attack, vascular disease, dyslipidaemia, prior bleeding, alcohol use disorder, renal failure, liver cirrhosis or failure, cancer, dementia, psychiatric disorders, and income level (divided in tertiles). Additionally, in analyses, also including follow-up time with OAC use (i.e. the first approach described above covering the entire follow-up and the analyses on the 1-year follow-up after AF diagnosis), the adjusted analyses included also OAC use, with the follow-up time split according to OAC exposure. This exposure was considered to start from the first OAC purchase and continue until 120 days after the last drug purchase. The 120-day interval was chosen since in Finland it is possible to purchase drugs with reimbursement for a maximum of 90 days and an additional 30-day grace period was allowed to cover possible stockpiling and differences in warfarin dosing. Thereafter, we fitted the regression models with the interaction term between calendar year period and sex, to assess whether the association between sex and IS rate has changed over time. The χ^2^ test, Student’s *t*-test, and analysis of variance were used to compare baseline variables. Statistical analyses were performed with the IBM SPSS Statistics software version 28.0 (SPSS, Inc., Chicago, IL, USA) and R version 4.0.5 (R Core Team, Vienna, Austria; https://www.R-project.org).

## Results

We identified 229 565 patients with new-onset AF (50.0% women; mean age 72.7 years; mean follow-up time 4.0 years; for comparison, the overall adult population over 20 years of age in Finland in 2018 was 4.1 million). When compared with men, women were older and had higher burden of comorbidities and lower income, which were reflected also in their higher stroke risk scores (*Table 1*). The mean age at the time of AF diagnosis increased over time, particularly in men. Likewise, prevalence of comorbidities and the baseline stroke risk scores increased during the study period in both men and women, whereas the income disparities remained similar across the study period (see Supplementary data online, *Table S2*). Oral anticoagulant was initiated in 71.5% of women and 69.5% of men during follow-up. One-year mortality after the diagnosis of AF decreased continuously from 13.1% to 8.7% during the study period. The decrease was observed both in men and women, and mortality was constantly higher in women than in men (see Supplementary data online, *Table S3*).

**Table 1:** Characteristics of the study cohort

	Women	Men	*P*-value
	*n* = 114 823	*n* = 114 747	
**Mean age at baseline, years**	76.6 (11.8)	68.9 (13.4)	<.001
**Income tertiles**			<.001
1st (lowest)	52 484 (46.0)	25 403 (22.1)	
2nd	38 162 (33.2)	38 162 (31.8)	
3rd (highest)	23 813 (20.7)	52 858 (46.1)	
**Highest education level**			<.001
Primary school	67 816 (59.1)	52 561 (45.8)	
Upper secondary education	28 091 (24.5)	33 900 (29.5)	
Higher education	18 916 (16.5)	28 281 (24.6)	
**Baseline comorbidities**			
Any vascular disease	30 970 (27.0)	33 379 (29.1)	<.001
Diabetes	23 764 (20.7)	25 783 (22.5)	<.001
Dyslipidaemia	56 321 (49.1)	53 331 (46.5)	<.001
Heart failure	22 199 (19.3)	17 718 (15.4)	<.001
Hypertension	92 288 (80.4)	77 966 (67.9)	<.001
Prior IS or TIA	19 267 (16.8)	16 108 (14.0)	<.001
Abnormal liver function	567 (0.4)	693 (0.6)	<.001
Abnormal renal function	4102 (3.6)	5028 (4.4)	<.001
Alcohol use disorder	1836 (1.6)	7226 (6.3)	<.001
Cancer	25 359 (22.1)	21 893 (19.1)	<.001
Dementia	7638 (6.7)	4140 (3.6)	<.001
Prior bleeding	10 442 (9.1)	14 097 (12.3)	<.001
Psychiatric disorder	15 342 (13.4)	15 656 (13.6)	.05
**Risk scores**			
Mean modified HAS-BLED score	2.6 (0.9)	2.4 (1.1)	<.001
Mean CHA_2_DS_2_-VASc score	4.2 (1.6)	2.6 (1.8)	<.001
**Stroke risk categories**			<.001
Low stroke risk	4926 (4.3)	14 205 (12.4)	
Moderate stroke risk	12 277 (10.7)	20 262 (17.7)	
High stroke risk	97 620 (85.0)	80 275 (70.0)	
**OAC use during follow-up**			
Any OAC	82 112 (71.5)	79 743 (69.5)	<.001
DOAC	25 414 (22.1)	26 743 (23.3)	<.001

The values represent counts (percentages) or means (standard deviations).

CHA_2_DS_2_-VASc score, congestive heart failure (1 point), hypertension (1 point), age ≥ 75 years (2 points), diabetes (1 point), history of stroke or TIA (2 points), vascular disease (1 point), age 65–74 years (1 point), and sex category (female) (1 point); DOAC, direct oral anticoagulant; IS, ischaemic stroke; modified HAS-BLED score, hypertension (1 point), abnormal renal or liver function (1 point each), prior stroke (1 point), bleeding history (1 point), age > 65 years (1 point), alcohol abuse (1 point), and concomitant antiplatelet/non-steroidal anti-inflammatory drugs (NSAIDs) (1 point) [no labile international normalized ratio (INR), max score 8]; TIA, transient ischaemic attack. Stroke risk categories based on CHA_2_DS_2_-VASc score: low, 0 in men and 1 in women; moderate, 1 in men and 2 in women; and high, >1 in men and >2 in women; OAC, oral anticoagulant.

**Figure 1:**
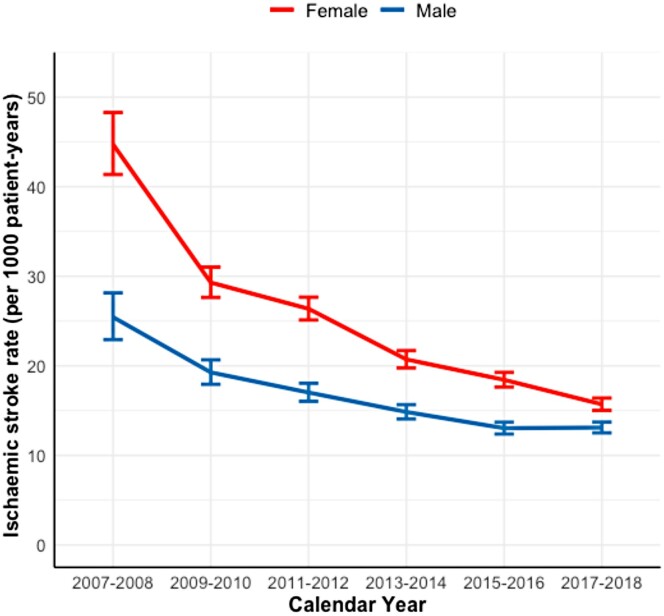
Trends in the crude ischaemic stroke rates with 95% confidence intervals in the entire follow-up

**Figure 2:**
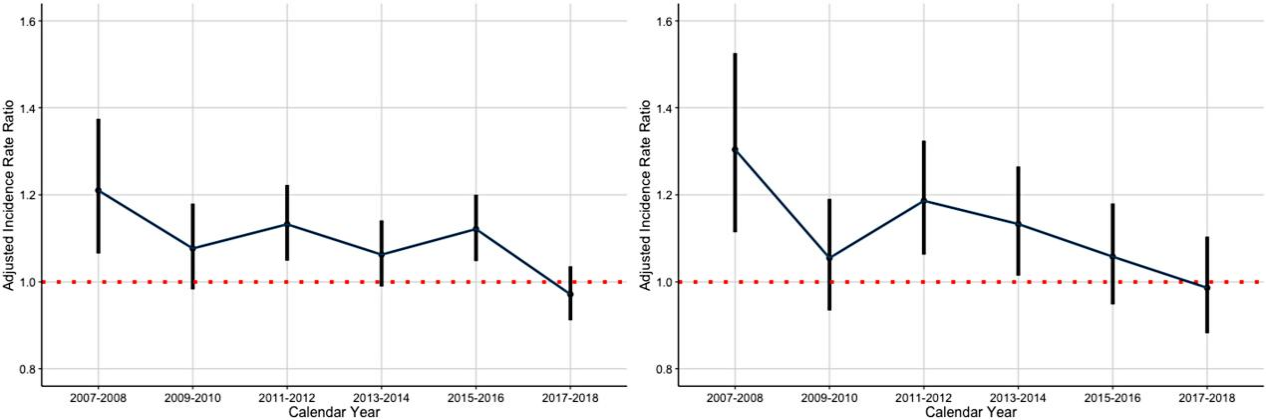
Adjusted incidence rate ratios of ischaemic stroke with 95% confidence intervals comparing women with men (red broken line) in the entire follow-up (left panel) and when considering only time without oral anticoagulant use (right panel)

**Figure 3:**
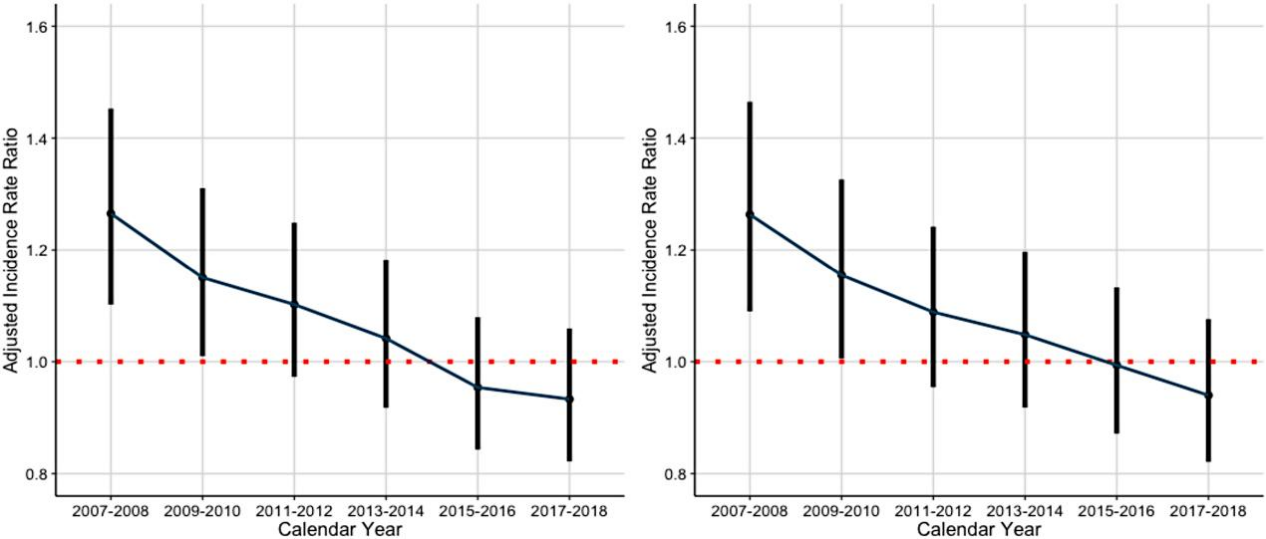
Adjusted incidence rate ratios of ischaemic stroke with 95% confidence intervals within a 1-year follow-up after atrial fibrillation diagnosis comparing women with men (red broken line) in the overall cohort (left panel) and in patients with high stroke risk (CHA_2_DS_2_-VASc score > 1 in men and >2 in women; right panel)

Crude IS rates decreased considerably over the study period, particularly in women; however, the rates remained consistently higher in women (*Figure 1*). Female sex was associated with a higher IS rate in the unadjusted analyses, but not after adjusting for confounding factors. Similar associations emerged when considering the entire follow-up period, as well as when examining only periods without OAC use (*Table 2*). We observed an interaction between the calendar year period and sex (interaction term *P* < .001). There was a significant association between female sex and IS rate at the start of the study period. However, thereafter, an overall decreasing pattern in this association was observed, eventually becoming statistically non-significant by the end of the observation period. Notably, non-linear variability was observed in this decline. A similar attenuation of the stroke risk associated with female sex was observed in the analyses with and without OAC use (*Figure 2*). In the analysis focusing solely on a 12-month follow-up period following the diagnosis of AF, a comparable, yet more linear, decline in the association between female sex and the risk of IS was observed (*Figure 3*). In the sensitivity analyses covering only patients with AF diagnoses in the hospital setting, the findings were uniform to those of the analyses covering the overall cohort (see Supplementary data online, *Figure S3*).

**Table 2:** Incidence of ischaemic stroke in men and women from 2007 to 2018

	Events	Patient-years (1000 years)	Incidence (per 1000 patient-years)	Unadjusted IRR	Adjusted IRR
**Entire follow-up**					
Men	6999	470	14.9 (14.5–15.2)	(Reference)	(Reference)
Women	9297	441	21.1 (20.7–21.5)	1.42 (1.37–1.46)	1.02 (0.99–1.06)
**Follow-up without OAC use**					
Men	2986	174	17.2 (16.6–17.8)	(Reference)	(Reference)
Women	4136	141	29.4 (28.5–30.3)	1.71 (1.64–1.80)	1.05 (0.99–1.10)

95% confidence intervals in parenthesis. IRRs estimated by Poisson regression. Adjusted for calendar year period, age, hypertension, diabetes, heart failure, prior ischaemic stroke or transient ischaemic attack, vascular disease, prior bleeding, alcohol use disorder, renal failure, liver cirrhosis or failure, cancer, dementia, psychiatric disorders, and income level and OAC use.

IRR, incidence rate ratio; OAC, oral anticoagulant.

### One-year outcomes by risk category

When analyses were stratified by stroke risk categories, female sex was associated with IS rate within the 1-year follow-up in the high-risk patient group (CHA_2_DS_2_-VASc score > 1 in men and >2 in women; Supplementary data online, *Table S4*). Similar to the analyses of the overall cohort, a significant interaction between the calendar year period and sex was observed in the high-risk group (*P* < .001), and the initially observed higher IS risk associated with female sex linearly decreased and became non-significant over the study period (*Figure 3*). These findings were reiterated also in patients classified as having a very high stroke risk (CHA_2_DS_2_-VASc score > 2 in men and >3 in women; Supplementary data online, *Figure S2*). In patients with low or moderate IS risk at baseline, no association between female sex and IS rate was observed (see Supplementary data online, *Table S4*). In these risk categories, the interaction terms between the calendar year period and sex were non-significant (*P* = .84 for low risk and *P* = .87 for moderate risk), indicating the absence of significant temporal changes (see Supplementary data online, *Figures S4* and *S5*).

## Discussion

This nationwide retrospective cohort study demonstrated that the IS risk independently associated with female sex in patients with AF has decreased over time during the study period from 2007 to 2018 (*Structured Graphical Abstract*). Initially, female sex was associated with a 20%–30% higher risk of IS in 2007–08, but by the end of the study period, this association became non-significant. When patients were stratified by stroke risk categories, a decline in IS risk associated with female sex was observed in more contemporary high-risk patients, becoming non-significant between females and males.

To the best of our knowledge, this is the first study to investigate temporal trends in the IS risk associated with female sex in patients with AF. Moreover, while the interplay between sex and IS risk has been extensively researched, the linkage of data from all national healthcare registries covering uniquely every level of care enables us to conduct substantially more robust and unbiased analyses than prior studies on this topic. Of note, the CHA_2_DS_2_-VASc score was originally generated from the Euro Heart Survey sample of only ∼1000 patients with AF during 2003 and 2004 treated at hospital level.^2^ Likewise, subsequent larger observational studies exploring sex differences in stroke risk have relied on selected study samples from hospital-level data, predominantly from the early 2000s, and without accounting for possible confounding by socioeconomic factors.^6,27^ Thus, previous studies linking IS to female sex may have been susceptible to some selection, information, and confounding biases. Moreover, heterogeneity has been noted in the effect sizes among studies reporting the IS risks associated with female sex, reflecting differences in study designs and populations studied.^6,27^

We found that the crude IS rates decreased considerably during the study period, with a more pronounced decline observed among women. Although prior studies have not specifically addressed gender differences in IS trends among patients with AF, our findings are consistent with previous studies reporting improving prognosis of AF and decreasing overall stroke rates.^28–31^ Likewise, the magnitude of the observed decline in the overall IS rate is comparable with a Swedish study reporting a 42% decrease in IS rate from 2012 to 2018.^30^ In the beginning of the study period, female sex was associated with a 20%–30% higher IS rate, which corresponds with the effect sizes reported in previous meta-analyses.^6,27^ However, importantly, we observed that this association became non-significant with no difference between males and females in the more contemporary period, a finding previously unreported in any study.

Indeed, the association between female sex and IS rate exhibited an overall decreasing pattern in all of the adjusted analyses, although notably there was some variability in the analyses with longer follow-up times (*Figure 2*), as compared with the more linear trends observed in the analyses restricted to 1-year follow-up (*Figure 3*). The observed variability may thus stem from differences in follow-up duration, or represent natural fluctuations in IS incidence, or potentially be indicative of statistical fluctuation in the adjusted regressions. The attenuation in the association between female sex and IS was driven by the declining trends within the high stroke risk patient group, while in the low- and moderate-risk categories, female sex was not associated with a higher IS risk (see Supplementary data online, *Table S4*, *Figure 3*, and Supplementary data online, *Figures S3*–*S5*).

Taken together, the limited sex differences in IS amongst contemporary AF populations shown by the current FinACAF study cohort suggest that female sex could potentially be omitted as risk modifier when considering stroke risk stratification of patients with AF. Nevertheless, women tended to be under-treated with OACs in various observational studies and they sustain more severe IS events when they happen.^32–35^ Hence, consideration of female sex in stroke risk stratification might still draw attention to the potentially increased AF-related stroke risks in women. However, we have previously reported a resolution of gender inequalities in OAC use over time, and correspondingly, sex-related disparities in OAC utilization have not been evident in more contemporary registries, as compared with older ones, such as the Euro Heart Survey.^35–40^

Analogous to the causes underlying the initial IS risk associated with female sex, the decline in this association is most likely related to several factors, many of which are not captured by the current data. The comprehensive nationwide data enable more thorough adjustments than most previous studies, but since the magnitude of the initial IS risk linked to female sex aligns with earlier findings, our results are unlikely to be attributable to differences in controlling for confounding factors compared with prior research. Thus, the trend of declining impact of female sex on stroke risk is most probably linked to other concomitant health trends, such as improvements in risk factor management and lifestyle-related factors. Indeed, there are reports suggesting diminishing gender inequalities in various aspects of health, although clear disparities still persist.^41^ Likewise, advances made in reducing gender inequalities in wider societal contexts, such as in socioeconomic conditions, may also contribute to the findings of this study.^42^ The use of OACs in patients with AF has increased substantially over the past decade.^18,43^ However, since we observed the decrease in the risk of IS associated with female sex also in the analyses that considered only the time when OACs were not used, this trend seems not to be exclusively linked to the improved utilization of OAC therapy. The prevalence of diagnosed comorbidities increased in both men and women during the study period, which may reflect increased screening for comorbidities and more holistic management of patients with AF, which has been associated with improved clinical outcomes, including a lowered risk of stroke.^44,45^ Moreover, previous studies have noted that part of the higher IS risk in women is linked to inequalities in cardiovascular care, and although the prevalence of comorbidities increased in both genders in our study, decreasing inequalities in IS risk factor management may in part explain the findings of our study.^13^

### Strengths and limitations

A particular strength of the current study is the nationwide coverage of patients diagnosed with AF from all levels of care, mitigating selection bias and therefore improving the generalizability of the results.^17^ Additionally, the hospital care register used to define the IS events is well-validated and has relatively high diagnostic accuracy, especially regarding cardiovascular diseases.^46^ Nevertheless, the limitations of our study need to be acknowledged, the most important of which are the challenges inherent in register-based retrospective cohort studies. Thus, information bias may be present in the used administrative data, potentially impacting the accuracy of recorded comorbidities and outcome events. We only considered IS events from the validated hospital care register to ensure the validity of the event, and although practically all patients with a suspected IS are examined in a hospital setting in Finland, some events may have been missed with this approach. Moreover, our results reflect associations and not necessarily causation between sex and IS. Our study comprehensively covers patients with AF in Finland, yet further research is needed to investigate whether similar trends have occurred in other countries. Except for diagnosed alcohol use disorders, the administrative data used lacked information about lifestyle-related factors. Additionally, we lacked data on the subtype of AF as well as data on adherence to OAC therapy. Moreover, we focused on event rates rather than risks since the current guidelines are mainly based on estimation of expected IS rates.^10,47,48^ Finally, although the linked registry data allowed us to adjust the regressions for a vast number of potentially influencing factors, the possibility of residual confounding by other unmeasured factors cannot be excluded, such as incident comorbidities and drug therapy changes.

## Conclusion

In conclusion, the association between female sex and IS rate has decreased over time and become non-significant over the course of the study period from 2007 to 2018, suggesting that female sex could perhaps be omitted as a factor when estimating expected IS rates and the need for OAC therapy in patients with AF.

## Supplementary data

Supplementary data are available at *European Heart Journal* online.

## Supplementary Material

ehae198_Supplementary_Data

## Declarations

### Data Availability

Because of the sensitive nature of the data collected for this study, requests to access the dataset from qualified researchers trained in human subject confidentiality protocols may be sent to the Finnish national register holders (KELA, Finnish Institute for Health and Welfare, Population Register Center and Tax Register) through Findata (https://findata.fi).

### Pre-registered Clinical Trial Number

None supplied.
