# Supplementary material for: Ischaemic stroke in women with atrial fibrillation: temporal trends and clinical implications
Source: Eur Heart J. 2024 Apr 12;45(20):1819–27. doi: 10.1093/eurheartj/ehae198 (PMC11129795; doi:10.1093/eurheartj/ehae198)

**Supplementary Material**

**Supplementary Table 1.** Definitions of the comorbidities

**Supplementary Table 2.** Baseline age and risk scores according to the calendar year intervals

**Supplementary Table 3.** Mortality within one year follow-up according to the calendar year intervals

**Supplementary Table 4.** Incidence of ischemic stroke in men and women according to stroke risk categories within the one year follow-up from the initial diagnosis of AF from 2007 to 2018 **Supplementary Figure 1.** Flow-chart of the patient selection process

**Supplementary Figure 2.** Adjusted incidence rate ratios of ischemic stroke with 95% confidence intervals within a one-year follow-up after AF diagnosis comparing women to men (red line) in patients with very high stroke risk (CHA_2_DS_2_-VASc score >2 in men and >3 in women)

**Supplementary Figure 3.** Adjusted incidence rate ratios of ischemic stroke with 95% confidence intervals comparing women to men (red line) covering only patients with AF identified from the hospital care register

**Supplementary Figure 4.** Adjusted incidence rate ratios of ischemic stroke with 95% confidence intervals within a one-year follow-up after AF diagnosis comparing women to men (red line) in patients with low risk (CHA_2_DS_2_-VASc = 0 in men and 1 in women)

**Supplementary Figure 5.** Adjusted incidence rate ratios of ischemic stroke with 95% confidence intervals within a one-year follow-up after AF diagnosis comparing women to men (red line) in patients with moderate risk (CHA_2_DS_2_-VASc = 1 in men and 2 in women)

**Supplementary Table 1**. Definitions of the comorbidities

|  | ICD-10 | ICPC-2 | Reimbursement code | ATC code | Other |
| --- | --- | --- | --- | --- | --- |
| Any vascular disease | I20-I25, I65-I66, I67.2, I70 | K74, K75, K76, K91, K92 | 206 |  |  |
| Hypertension | I10-I15 | K85, K86, K87 | 205 | C03A, C03B, C03DB, C03EA, C07A, C08CA, C08D, C09 |  |
| Dyslipidemia | E78 | T93 | 206 | C10 |  |
| Heart failure | I50, I11.0, I13.0, I13.2 | K77 | 201 |  |  |
| Diabetes | E10-E14 | T89, T90 | 103, 215 | A10 |  |
| Previous stroke | I63, I64, I69.3-I69.8 | K90 |  |  |  |
| Bleeding history | D50.0, D62, D68.3, I60-I62, I69.0-I69.2, I85.0, I86.4, J94.2, K22.1, K22.3, K22.6, K25.0, K25.2, K25.4, K25.6, K26.0, K26.2, K26.4, K26.6, K27.0, K27.2, K27.4, K27.6, K28.0, K28.2, K28.4, K28.6, K29.0, K62.5, K63.1, K63.3, K92.0-K92.2, N02, R04, R31, R58, S06.2-S06.6, S06.8 |  |  |  |  |
| Alcohol abuse | F10 |  |  |  |  |
| Renal failure or dialysis | N18, Z49 |  |  |  |  |
| Liver cirrhosis or failure | K70.2-K70.4, K71.7, K71.8, K72, K74 |  |  |  |  |
| Dementia | F00-F03, G30 |  |  |  |  |
| Cancer |  |  |  |  | Any cancer registered in the Finnish Cancer Registry |
| Psychiatric disorder | F04-F99 |  |  |  |  |

Abbreviations: ATC, anatomic therapeutic chemical; ICD-10, International Classification of Diseases, Tenth Revision; ICPC-2, International Classification of Primary Care, Second Edition

**Supplementary Table 2.** Baseline demographics according to the calendar year intervals

|  | **2007-2008** | | | **2009-2010** | | **2011-2012** | | **2013-2014** | | **2015-2016** | | **2017-2018** | |
| --- | --- | --- | --- | --- | --- | --- | --- | --- | --- | --- | --- | --- | --- |
|  | **Men** | | **Women** | **Men** | **Women** | **Men** | **Women** | **Men** | **Women** | **Men** | **Women** | **Men** | **Women** |
| Number of patients | 16 307 | | 16 722 | 15 905 | 16 576 | 18 998 | 19 036 | 19 785 | 19 801 | 20 967 | 20 685 | 22 780 | 22 003 |
| Mean age (years) | 66.9 | | 76.2 | 67.5 | 76.0 | 68.5 | 76.5 | 69.0 | 76.3 | 69.9 | 76.8 | 70.7 | 77.2 |
| Mean CHA_2_DS_2_-VASc | 2.2 | | 4.0 | 2.4 | 4.1 | 2.5 | 4.2 | 2.7 | 4.3 | 2.8 | 4.3 | 2.9 | 4.4 |
| Mean HAS-BLED | 1.9 | | 2.3 | 2.1 | 2.4 | 2.3 | 2.6 | 2.4 | 2.7 | 2.6 | 2.8 | 2.7 | 2.9 |
| Any vascular disease | 25.9 | | 26.3 | 27.7 | 27.0 | 28.6 | 27.2 | 29.6 | 27.4 | 30.0 | 26.9 | 31.5 | 27.0 |
| Diabetes | 16.0 | | 16.4 | 18.8 | 17.4 | 20.8 | 19.4 | 23.0 | 21.7 | 26.0 | 23.2 | 27.3 | 24.3 |
| Heart failure | 14.8 | | 19.5 | 16.6 | 21.0 | 15.4 | 19.8 | 15.7 | 19.8 | 15.3 | 18.5 | 15.1 | 17.9 |
| Hypertension | 59.0 | | 74.0 | 62.3 | 77.5 | 67.1 | 80.3 | 69.0 | 81.7 | 72.3 | 82.4 | 74.1 | 84.1 |
| Prior IS or TIA | 10.6 | | 13.6 | 12.0 | 15.5 | 13.6 | 16.0 | 14.5 | 17.4 | 15.7 | 18.4 | 16.4 | 18.7 |
| **Stroke risk categories** | |  |  |  |  |  |  |  |  |  |  |  |  |
| Low stroke risk | 17.8 | | 5.7 | 15.4 | 5.5 | 12.8 | 4.5 | 11.7 | 4.2 | 10.2 | 3.6 | 8.6 | 2.8 |
| Moderate stroke risk | 20.9 | | 11.8 | 20.2 | 12.1 | 18.6 | 11.0 | 17.4 | 10.5 | 15.5 | 9.7 | 15.0 | 9.6 |
| High stroke risk | 61.3 | | 82.5 | 64.4 | 82.4 | 68.6 | 84.5 | 70.9 | 85.3 | 74.3 | 86.7 | 76.4 | 87.6 |
| **Income tertiles** |  | |  |  |  |  |  |  |  |  |  |  |  |
| 1^st^ (lowest) | 22.3 | | 44.3 | 21.6 | 45.7 | 20.8 | 44.9 | 22.5 | 46.6 | 21.6 | 45.1 | 22.4 | 45.4 |
| 2^nd^ | 31.1 | | 34.5 | 31.6 | 33.1 | 33.7 | 35.1 | 31.9 | 33.0 | 32.6 | 35.1 | 31.9 | 34.0 |
| 3^rd^ (highest) | 46.6 | | 21.3 | 46.8 | 21.3 | 45.5 | 20.0 | 45.6 | 20.4 | 45.8 | 19.8 | 45.7 | 20.6 |
| The values represent percentages unless otherwise specified. All sex differences within calendar year intervals p<0.001. Difference in any vascular disease between calendar year intervals among women p = 024. Test for linearity in any vascular disease among women p = 0.33, test for linearity in heart failure among men p = 0.26 and test for linearity in income tertiles among men p= 0.06 and among women p=0.05. All other differences between calendar year intervals both in men and women separately p<0.001 and tests for linearity both in men and women in all other variables p<0.001. CHA_2_DS_2_-VASc score, congestive heart failure (1 point), hypertension (1 point), age ≥75 years (2 points), diabetes (1 point), history of stroke or TIA (2 points), vascular disease (1 point), age 65-74 years (1 point), sex category (female) (1 point); modified HAS-BLED score, hypertension (1 point), abnormal renal or liver function (1 point each), prior stroke (1 point), bleeding history (1 point), age >65 years (1 point), alcohol abuse (1 point), concomitant antiplatelet/NSAIDs (1 point) (no labile INR, max score 8). | | | | | | | | | | | | | |

**Supplementary Table 3.** Mortality within one year follow-up according to the calendar year intervals

| **2007-2008** | | **2009-2010** | | **2011-2012** | | **2013-2014** | | **2015-2016** | | **2017-2018** | | |
| --- | --- | --- | --- | --- | --- | --- | --- | --- | --- | --- | --- | --- |
| **Men** | **Women** | **Men** | **Women** | **Men** | **Women** | **Men** | **Women** | **Men** | **Women** | **Men** | **Women** | |
| 11.8% | 14.4% | 11.5% | 14.1% | 10.6% | 12.3% | 10.1% | 12.0% | 10.1% | 11.6% | 8.4% | 9.0% | |
| All sex differences within calendar year intervals p<0.001. All differences between calendar year intervals both in men and women separately p<0.001 and tests for linearity both in men and women in all variables p<0.001. | | | | | | | | | | | |  |

**Supplementary Table 4.** Incidence of ischemic stroke in men and women according to stroke risk categories within the one year follow-up from the initial diagnosis of AF from 2007 to 2018

|  | **Events** | **Patient years (1000 years)** | **Incidence (per 1000 patient years)** | **Unadjusted IRR** | **Adjusted IRR** |
| --- | --- | --- | --- | --- | --- |
| **Low risk** |  |  |  |  |  |
| Men | 114 | 13 | 8.5 (7.0-10.2) | (Reference) | (Reference) |
| Women | 33 | 5 | 7.0 (4.8-9.9) | 0.83 (0.56-1.22) | 0.74 (0.50-1.10) |
| **Moderate risk** | |  |  |  |  |
| Men | 247 | 19 | 13.2 (11.6-14.9) | (Reference) | (Reference) |
| Women | 175 | 11 | 15.2 (13.1-17.7) | 1.16 (0.95-1.41) | 1.10 (0.90-1.34) |
| **High risk** |  |  |  |  |  |
| Men | 2 159 | 68 | 31.8 (30.4-33.1) | (Reference) | (Reference) |
| Women | 3 181 | 82 | 38.6 (37.3-38.8) | 1.22 (1.15-1.28) | 1.07 (1.01-1.14) |
| Abbreviations: IRR, incidence rate ratio. 95% confidence intervals in parenthesis. IRRs estimated by Poisson regression. Adjusted for calendar year period, age, hypertension, diabetes, heart failure, prior ischemic stroke or transient ischemic attack and vascular disease, prior bleeding, alcohol use disorder, renal failure, liver cirrhosis or failure, cancer, dementia, psychiatric disorders, income level, and oral anticoagulant use. Stroke risk categories based on CHA_2_DS_2_-VASc score: low, 0 in men and 1 in women; moderate, 1 in men and 2 in women; high, >1 in men and >2 in women. | | | | | |

**Supplementary Figure 1.** Flow-chart of the patient selection process


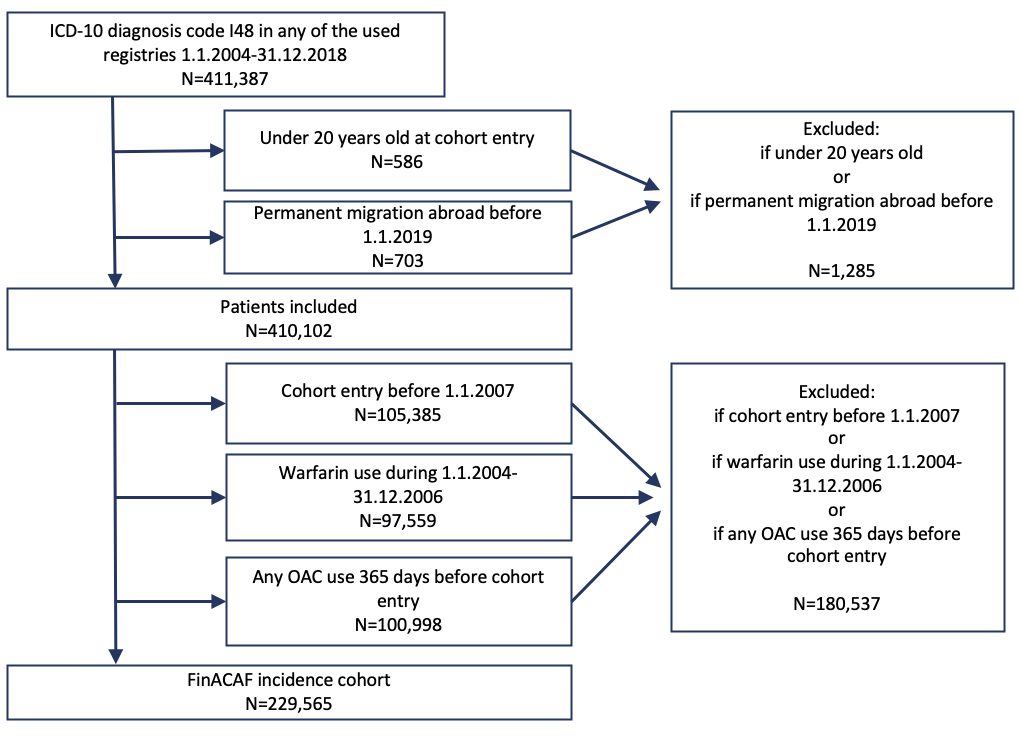


**Supplementary Figure 2.** Adjusted incidence rate ratios of ischemic stroke with 95% confidence intervals within a one-year follow-up after AF diagnosis comparing women to men (red line) in patients with a very high stroke risk (CHA_2_DS_2_-VASc score >2 in men and >3 in women)


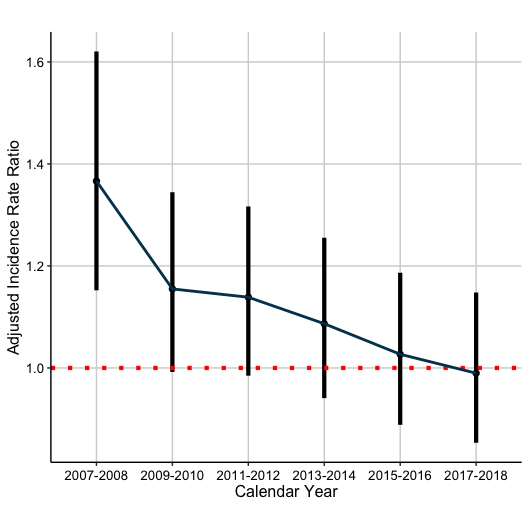


**Supplementary Figure 3.** Adjusted incidence rate ratios of ischemic stroke with 95% confidence intervals comparing women to men (red line) covering only patients with AF identified from the hospital care register.


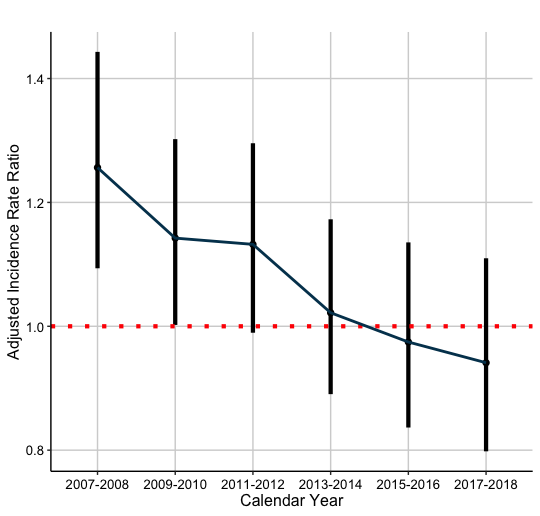


**Supplementary Figure 4.** Adjusted incidence rate ratios of ischemic stroke with 95% confidence intervals within a one-year follow-up after AF diagnosis comparing women to men (red line) in patients with low risk (CHA_2_DS_2_-VASc = 0 in men and 1 in women)


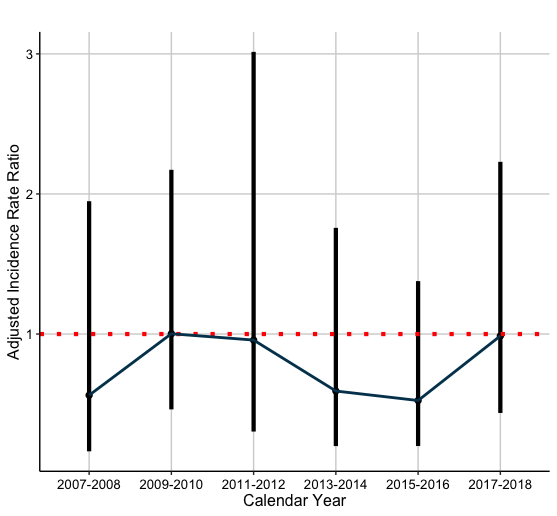


**Supplementary Figure 5.** Adjusted incidence rate ratios of ischemic stroke with 95% confidence intervals within a one-year follow-up after AF diagnosis comparing women to men (red line) in patients with moderate risk (CHA_2_DS_2_-VASc = 1 in men and 2 in women)


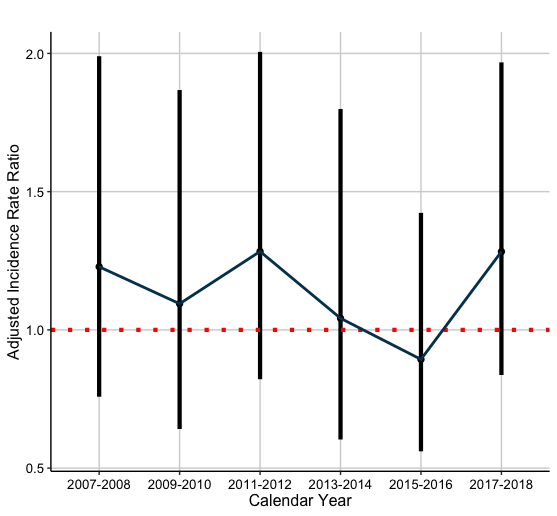

Supplement: ehae198_Supplementary_Data [file ehae198_supplementary_data.docx]
